# Merkel Cell Carcinoma and Diagnostic Experience in a Reference Hospital: A Case Series

**DOI:** 10.1155/2020/8391510

**Published:** 2020-02-19

**Authors:** Rocio del Pilar López Panqueva, David A. Suarez-Zamora, Luis E. Barrera-Herrera, Mariam Rolón Cadena

**Affiliations:** ^1^Department of Pathology and Laboratories, Fundación Santa Fe de Bogotá, Bogotá, Colombia; ^2^Faculty of Medicine, Universidad de Los Andes, Bogotá, Colombia

## Abstract

Merkel cell carcinoma (MCC) is a rare poorly differentiated neuroendocrine tumor, usually located in sun-exposed skin, with aggressive behavior and with high recurrence risk and metastatic disease. In Latin America, case series have been published, and it does not exceed 32 patients in 10 years, and in Colombia, there are case reports. We present a descriptive retrospective cross-sectional study in patients diagnosed with MCC in the Department of Pathology and Laboratories at the University Hospital Fundación Santa Fe de Bogotá(FSFB) between January 2003 and December 2018. We present the demographic, clinical, and pathological variables of these patients, as well as a literature review.

## 1. Introduction

Merkel cell carcinoma (MCC) is a rare and aggressive cutaneous malignancy with unknown cellular origin, and current information reveals a trilinear differentiation; epithelial, neuroendocrine, and early B cell lymphocytic carcinoma [1]. 5-year survival for advanced-stage MCC has been reported between 0 and 18% [1], the incidence of Merkel cell carcinoma is about 3 per 1000000 worldwide, and incidences appear to be increased either due to an aging population or improved detection [2]. MCC incidence varies by geographic regions, and it ranges from 0.13 per 100000 person-years in Europe to 1.6 per 100000 person-years. It accounts for <1% of cutaneous cancers in Europe based on a retrospective cohort study with 20 diagnosed MCC cases. In Colombia and Latin America, there are some reports published; one of the biggest reports is from Carneiro et al. with 32 cases presented in a period of 10 years in the Instituto Nacional de Cancer, on which they report late diagnosis that leads to high lethality by invasive disease [3].

Many reports of case series have identified the clinical features of MCC, and typically it present as a firm, painless red-violet nodule that is dome-shaped and has undergone rapid growth [4, 5]. Nodules are common in areas exposed to sun, most frequently occur on head and neck, and less common on lower limbs, trunk, or other areas not exposed to sunlight [6]. History of extensive sun/ultraviolet (UV) exposure is recognized as a likely risk factor, and others include older age (most patients > 65 years old), Caucasian ethnicity, polyomavirus infection (reported 45%–100% of cases of MCC), and immunosuppressive states [2, 6, 7]. Some authors have described that Merkel cell polyomavirus (MCPyV) positive displayed very low mutation rates, but MCC polyomavirus negative exhibited a high mutation burden associated with a UV-induced DNA damage signature [8]. UV-induced damage is associated with the anatomic site of presentation and could differ by sex from the information of population surveillance [9].

For the definitive diagnosis, a biopsy is required; histology of MCC typically shows hyperchromatic nuclei and high mitotic activity on hematoxylin and eosin (H & E) staining. Histologic subtypes include intermediate type-most common, characterized by basophilic nuclei with high mitotic activity; trabecular type-rare usually only seen in mixed tumors; and small cell type-undifferentiated and may be identical to small-cell carcinomas of other sites, and less common morphological subtypes include glandular, squamous, and melanocytic carcinomas [1]. An immunohistochemistry study has to be performed with the appropriated immunopanel, and cytokeratin 20 (CK-20) was reported to be positive in 89%–100% of cases of MCC and considered testing for other neuroendocrine tumor markers to exclude other differential diagnoses including chromogranin A and synaptophysin.

## 2. Materials and Methods

A retrospective review of all pathology samples with diagnosis of MCC and/or poorly differentiated neuroendocrine carcinoma in the skin was conducted in the University Hospital Fundación Santa Fe de Bogotá between January 2003 and December 2018. Approval from the Institutional Review Board was obtained before data collection. Data were extracted from the electronic medical record, and each case was compared to electronic pathology records to find pathological characteristics reported.

The medical information was reviewed and compared to the reported in the pathology database for each analyzed case, and then each pathology slide was reviewed and aditional clinically relevant information was included in a medical independent database designed for this investigation. Cases found in the database and identify as repeated were excluded. 

## 3. Results

**Figure 1:**
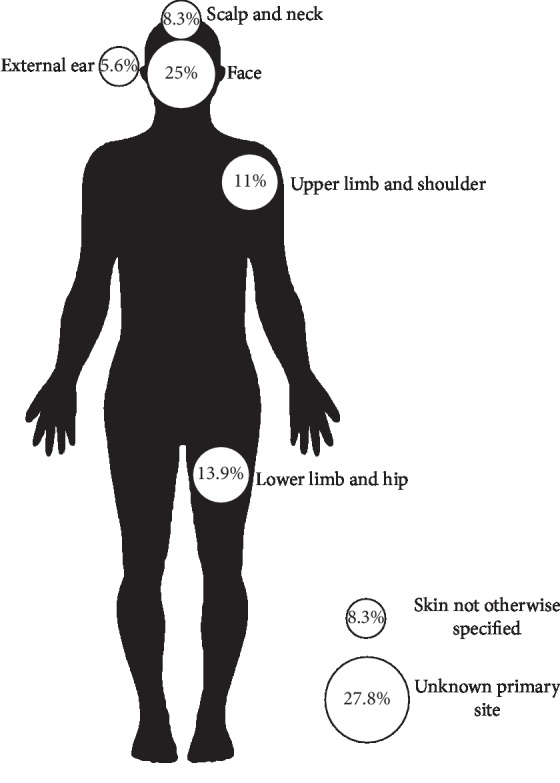
Tumor localization.

A total of 36 patients included in the review during the 10 years were analyzed in our pathology lab; there were 12 men (55.6%) and 16 women (44.4%), with a median age of 68 years (range 17–87 years). Clinicopathological characteristics of MCC are shown in Table 1 and Figure 1; the head and neck were the most common locations. 41% followed by the primary site were unknown and the last was skin without other specification (8.3%). Cytokeratin 7 was positive in 2 patients (5.6%), cytokeratin 20 was positive in 33 patients (91.1%), chromogranin A was positive in 25 patients (83.3%), and synaptophysin was positive in 24 patients (96.0%).

**Figure 2:**
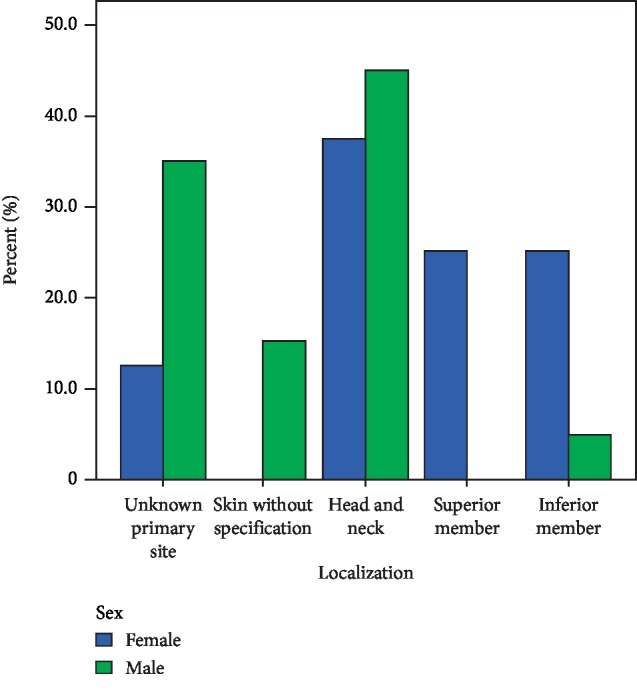
Distribution by sex.

We analyzed the localization by sex (Tables 2 and 3), and it showed that unknown primary site was 2 (12.5%) in female and 7 (35%) in male, skin without specification was 0 in women and 3 (15%) in men, head and neck localization was 6 (37.5%) in women and 9 (45%) in men; upper limb 4 (25%) in women and 0 in men, and lower limb in women was 4 (25%) and 1 (5%) in men.  Figure 2 shows the distribution of localization by sex. We match the CK-20 results with neuroendocrine markers; CK-20 was positive in 30 of the neuroendocrine markers (93.8%), and 2 CK-20 positive were negative for neuroendocrine markers. 2 samples negative for CK-20 were positive for neuroendocrine markers and one sample did not have CK-20 result.

## 4. Discussion

Merkel cell carcinoma is a rare skin tumor and its diagnosis is difficult. The diagnosis of Merkel cell carcinoma is confirmed with immunohistochemical stains for distinction from other small-cell malignancies, as small-cell lung cancer, melanoma, or lymphoma. CK-20 is a marker for MCC and is found positive in 89–100% of cases, other neuroendocrine markers that are used to support the diagnosis of MCC are synaptophysin and chromogranin, and others are positively stained by MCC [10]. This series of cases shows the experience of Merkel diagnosis in 16 years in a reference center of pathology.

This review shows the clinical presentation and the confirmatory immunohistochemical stains (Table 4) to differentiate from other neuroendocrine tumors. From this analysis, the head and neck were the most common localization, followed by primary site unknown, and this review similar to others reports of Hitchcock et al. who reported primary site lesion as head and neck 48.9%, upper extremities 15.6%, and lower extremities 30.2%, but without report of the unknown primary site [11]. Heath et al. reported the unknown primary site in 14% of 195 cases similar to this study [5], which is less in limbs due to reports of skin without other specifications in our study.

This review of 37 cases of diagnosis of MCC also showed a trend in limb localization for women, especially in lower limbs. 25% in upper limb and lower limb in women and 0% and 5%, respectively, in men. It differs from the study by Agelli et al. that reported similar presentations in limbs by sex in males (20.6% upper limbs and 17.5% lower limbs) and females (17.5% in upper limbs and 14.4% in lower limbs) [9]. These gender differences are not well explained in this study; we hypothesized that these differences could be possible due to exposure of limbs to sunlight in women in tropical countries because of wearing skirts. The association of gender and upper limbs are described in melanoma studies. Guiasvand et al. reported a stronger risk factor for melanoma on lower limbs (RR 3.38; 95% CI, 2.62–4.38) in Norwegian women [12]. Another explanation could be the relationship between positive MCPvV in female patients which is more prevalent than in male patients (66.7% vs 39.6) and MCPyV and MCC are more common in limbs [13]. However, there is not enough available information in Latin American countries to explain this finding.

## Figures and Tables

**Table 1:** Patient characteristics.

Patient ID	Age	Sex	Regional localization	Specific localization	CK7	CK20	Chromo.	Sinapt.
1	62	F	External ear	Auricular pavilion	−	+	+	+
2	81	M	External ear	Auricular pavilion	−	+	+	+
3	57	F	Skin face	Eyelid	ND	+	+	+
4	60	M	Skin face	Forehead	+	−	ND	+
5	64	F	Skin face	Cheekbone	−	+	ND	+
6	65	M	Skin face	Eyelid	−	+	+	ND
7	74	F	Skin face	Cheekbone	−	+	+	ND
8	75	M	Skin face	Cheekbone	ND	+	ND	+
9	76	F	Skin face	Cheekbone	ND	+	+	+
10	77	M	Skin face	Eyelid	ND	+	+	ND
11	84	M	Skin face	Forehead	−	+	+	+
12	86	M	Skin face	Forehead	ND	+	−	ND
13	17	F	Skin LLH	Finger	+	+	+	ND
14	56	F	Skin LLH	Thigh	−	+	+	+
15	73	F	Skin LLH	Thigh	−	+	+	+
16	83	F	Skin LLH	Thigh	ND	ND	ND	ND
17	86	M	Skin LLH	Thigh	ND	+	+	+
18	49	M	Skin NOS	—	−	+	+	ND
19	82	M	Skin NOS	—	−	+	+	ND
20	87	M	Skin NOS	—	ND	+	ND	ND
21	72	M	Skin ScNk	Neck	−	+	+	+
22	86	F	Skin ScNk	Scalp	−	+	+	+
23	87	M	Skin ScNk	Scalp	−	−	ND	+
24	32	F	Skin UPLS	Arm	−	+	+	ND
25	61	F	Skin UPLS	Arm	−	+	+	+
26	69	F	Skin UPLS	Arm	ND	+	+	+
27	74	F	Skin UPLS	Arm	−	+	ND	+
28	48	M	UNKPO	-	−	+	+	−
29	56	M	UNKPO	-	−	+	+	+
30	64	F	UNKPO	-	−	+	+	+
31	70	M	UNKPO	-	−	+	−	ND
32	71	M	UNKPO	-	−	−	+	+
33	71	M	UNKPO	-	−	+	+	+
34	71	M	UNKPO	-	−	+	−	+
35	76	M	UNKPO	-	−	+	+	+
36	81	F	UNKPO	-	ND	−	−	+

UNKPO, unknown primary origin; skin NOS, skin not otherwise specified; skin UPLS, skin, upper limb, and shoulder; skin LLH, skin, lower limb, and hip; skin ScNk, skin, scalp, and neck; ND, no data.

**Table 2:** Population.

Characteristics	*n* = 36
Sex	
Male	20 (55.6%)
Female	16 (44.4%)

Age, years	
Median	68.97
Range	17–87

Localization	
Primary site unknown	9 (25%)
Skin without other specification	3 (8.3%)
Head and neck	15 (41.7%)

Upper limb	4 (11.1%)
Lower limb	5 (13.9%)
Ck 7	Positive 2 (5.6%)
Ck-20	Positive 33 (91.1%)
Chromo./sinapt.	Positive 32 (88.9%)

**Table 3:** Localization by sex.

Localization	Sex	
	Female	Male
Unknown primary	2 (12.5%)	7 (35%)
Skin without specification	0 (0%)	3 (15%)
Head and neck	6 (37.5%)	9 (45%)
Superior member	4 (25%)	0 (0%)
Inferior member	4 (25%)	1 (5%)
Total	16	20

**Table 4:** Neuroendocrine markers vs. CK-20 status.

	Neuroendocrine markers	
	Negative	Positive
CK-20		
** **Negative	0	2 (6.2%)
** **Positive	2 (100%)	30 (93.8%)
Total	2	32
